# Milling the Mistletoe: Nanotechnological Conversion of African Mistletoe (*Loranthus micranthus*) Intoantimicrobial Materials

**DOI:** 10.3390/antiox7040060

**Published:** 2018-04-20

**Authors:** Muhammad Sarfraz, Sharoon Griffin, Tamara Gabour Sad, Rama Alhasan, Muhammad Jawad Nasim, Muhammad Irfan Masood, Karl Herbert Schäfer, Chukwunonso E.C.C. Ejike, Cornelia M. Keck, Claus Jacob, Azubuike P. Ebokaiwe

**Affiliations:** 1 Division of Bioorganic Chemistry, School of Pharmacy, Saarland University, D-66123 Saarbruecken, Germany; s8musarf@stud.uni-saarland.de (M.S.); sharoongriffin@gmail.com (S.G.); s8tagabo@stud.uni-saarland.de (T.G.S.); S8rralha@stud.uni-saarland.de (R.A.); jawadnasimpk@yahoo.com (M.J.N.); 2 Institute of Pharmaceutics and Biopharmaceutics, Philipps-Universität Marburg, 35037 Marburg, Germany; 3 Department of Biotechnology, University of Applied Sciences Kaiserslautern, 66482 Zweibruecken, Germany; s8mumaso@stud.uni-saarland.de (M.I.M.); karl-herbert.schaefer@hs-kl.de (K.H.S.); 4 Department of Medical Biochemistry, Federal University, Ndufu-Alike Ikwo, PMB 1010 Abakaliki, Nigeria; ejike.chukwunonso@funai.edu.ng; 5 Department of Chemistry/Biochemistry and Molecular Biology, Federal University, Ndufu-Alike Ikwo, PMB 1010 Abakaliki, Ebonyi State, Nigeria

**Keywords:** antimicrobial activity, mistletoe nanoparticle, *Loranthus micranthus*, nanosizing, nematicidal activity

## Abstract

Nanosizing represents a straight forward technique to unlock the biological activity of complex plant materials. The aim of this study was to develop herbal nanoparticles with medicinal value from dried leaves and stems of *Loranthus micranthus* with the aid of ball-milling, high speed stirring, and high-pressure homogenization techniques. The milled nanoparticles were characterized using laser diffraction analysis, photon correlation spectroscopy analysis, and light microscopy. The average size of leaf nanoparticles was around 245 nm and that of stem nanoparticles was around 180 nm. The nanoparticles were tested for their antimicrobial and nematicidal properties against a Gram-negative bacterium *Escherichia coli*, a Gram-positive bacterium *Staphylococcus carnosus*, fungi *Candida albicans* and *Saccharomyces cerevisiae*, and a nematode *Steinernemafeltiae*. The results show significant activities for both leaf and (particularly) stem nanoparticles of *Loranthus micranthus* on all organisms tested, even at a particle concentration as low as 0.01% (*w*/*w*). The results observed indicate that nanoparticles (especially of the stem) of *Loranthus micranthus* could serve as novel antimicrobial agents with wide-ranging biomedical applications.

## 1. Introduction

Recent advances in the highly multidisciplinary field of nanotechnology have also stimulated innovative ideas in drug design and delivery, eventually leading to the development of novel nanosized materials for biomedical, pharmaceutical, and agricultural applications [1,2]. These studies have been very timely, as there is currently an urgent need for new and innovative drugs in general, and antimicrobial agents in particular, especially in the developing world with its fast-growing population and still limited access to sufficient amounts of effective medicines.

**Figure 1:**
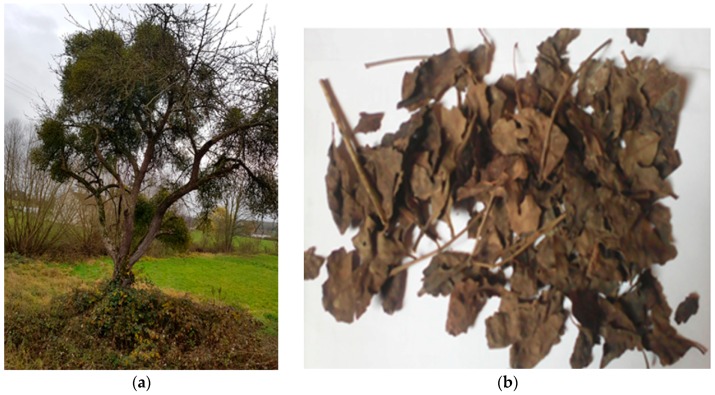
A live plant of mistletoe growing in a tree (**a**) and the dried leaves and stem of such a plant (**b**).

The African mistletoe (*Loranthus micranthus*), an obligate semi-parasitic plant of the family *Loranthaceae*, is common in the rain forest region of Nigeria and other parts of Africa, Europe, and America, where it grows primarily on *Kola accuminata, Baphianitida, Citrus limon,* and Pentacle thramacrophylla (Figure 1).

This plant, and its leaves and stems in particular, have a long tradition of being used in Folk Medicine, from the famous ancient Celtic druid Getafix (superfluous) to more realistic modern-day applications in herbal remedies and in the developing world [3,4]. Indeed, such applications are not entirely unfounded, as chemical screening and analyses have shown that *L*. *micranthus* contains lecithin, viscotoxin, polysaccharides, and many other biologically active phytochemicals [5]. The bioactive ingredients in the African mistletoe have been linked to its antimicrobial properties against a broad spectrum of pathogenic microorganisms, including bacteria and fungi [6]. Not surprisingly, extracts of this plant are frequently employed in practice, yet the production of the liquid extracts, fractions, and formulations requires certain equipment and adequate solvents, is time consuming, expensive, and also results in considerable waste.

We have therefore considered nanosizing as a possible alternative, as this method requires only a few steps from raw to final product. It can be applied globally and, by milling entire plants, maintains the chemical composition of the original material without adding any artificial ingredients or wasting any material [7,8].

## 2. Materials and Methods 

### 2.1. Production of Nanoparticles

The plants of *L*. *micranthus* were harvested in the forests near Federal University, Ndufu-Alike Ikwo, Abakaliki, Ebonyi State, the federal republic of Nigeria. The plants were identified botanically at the Department of Biology, Federal University, Ndufu-Alike Ikwo, Abakaliki, Ebonyi State, the federal republic of Nigeria, where a specimen of this plant had already been deposited prior to this study. The plants were separated into stem and leaves, and each plant part was dried immediately, in shade, at room temperature to reduce the moisture content for prolonged storage. The dried plant parts were blended into powder with a simple mechanical blender (household coffee grinder). The nanosizing of samples was conducted as described previously [9]. Briefly, each sample was initially reduced in size by dry milling. Bead milling was achieved using a FastPrep 24 Instrument (MP Biomedicals, Solon, OH, USA), with ceramic beads from Precellys Kits (Bertin Technologies, Montigny-le-Bretonneux, France). Subsequently, the samples were suspended in distilled water together with Plantacare^®^ 2000 UP (Base, Ludwigshafen, Germany) as 1% *w*/*w* coarse suspension. Each sample was then subjected to high speed stirring (HSS) with a Polytron^®^ PT2100 (Kinematica GmbH, Luzern, Switzerland) attached to a Polytron^®^ PT-DA-2112/EC aggregate. Three 1 min cycles of high speed stirring (HSS) were performed at 11,000 rpm. Subsequently, a high-pressure homogenizer (HPH) was employed as the so-called “premilling stage” in an APV Gaulin LAB40 high pressure homogenizer (APV GmbH, Mainz, Germany) with three consecutive cycles at 200, 500, and 1000 bar pressure and a final homogenisation cycle at 1500 bar pressure (1200 bar pressure was used in the case of the dried leaves).

### 2.2. Characterization of Nanoparticles

All steps for nanosizing were accompanied by adequate characterization procedures, according to a previous report (8). Laser Diffraction (LD) analysis with a Mastersizer 3000 (Malvern Instruments, Malvern, UK) and Photon Correlation Spectroscopy (PCS) with a Zetasizer Nano ZS (Malvern Instruments, Malvern, UK) were explored. This kind of analysis allows researchers to monitor the efficacy of each step and also to assess the response of the material to the different milling technologies employed. Furthermore, visual confirmation of the acquired nanosized material was determined by Light Microscopy (LM) using an Olympus BX53. Each analysis was repeated in triplicate. Results were represented as mean ± standard deviation (SD).

### 2.3. Assessment of Biological Activity

The antimicrobial and nematicidal activities of the nanosized suspensions were estimated in bacteria, including Gram-negative *Escherichia coli* and Gram-positive *Staphylococcus carnosus*, the pathogenic fungus *Candida albicans*, the yeast *Saccharomyces cerevisiae*, and a nematode with agricultural relevance, namely *Steinernema feltiae*.

#### 2.3.1. Determination of Antimicrobial Activity

Bacterial and fungal cell densities were transiently determined by measuring the optical density (OD) at 570 nm at 0 h (first measurement immediately after incubation), 4 h, and 24 h after incubation at 37 °C according to the method described by Zgoda and Porter [10] and using a Universal Microplate Reader, EL800, Biotech Instruments, Inc. Highland Park, Winooski, VT, USA. Nanoparticle suspensions of the samples were tested at final concentrations of 0.0125%, 0.025%, 0.05%, and 0.1% *w*/*w*. Nutrient *Luria-Bertanibroth* (LB), *bacterial basic media*, *Sabouraud Dextrose* (SD), and *Yeast Peptone Dextrose* (YPD) were used as media for *E. coli*, *S. carnosus*, *C. albicans*, and *S. cerevisiae*, respectively. The positive control was composed of a mixture of penicillin, streptomycin, and amphotericin B (4 U, 0.4 µg/mL, and 10 µg/mL, respectively) for *S. carnosus* and *E. coli*, while ketoconazole was used as the positive control for *C. albicans* and *S. cerevisiae*. The growth culture with medium for each organism was used as the negative control. The stabilizer 1% Plantacare^®^ stock was used as an additional, negative solvent control. All experiments were carried out in triplicate and on three different occasions (*n* = 9).

#### 2.3.2. Nematicidal Activity

The soil nematodes *S. feltiae* were purchased from Sautter and Stepper GmbH (Ammerbuch, Germany) and stored at 4 °C in the dark. Before each experiment, fresh samples of nematodes were purchased and stored in the freezer upon arrival and used for a maximum of six days. To perform the assay, 200 mg of nematodes was reconstituted in 50 mL of phosphate buffered saline (PBS) (pH = 7.4) to form a homogenous suspension to revive the nematodes. Afterwards, the individual suspensions were allowed to rest at room temperature. The sedimentation was avoided with occasional shaking in moderate light and at room temperature (25 °C) for 30 min. Viability of the nematodes was determined by counting the live and dead nematodes under a light microscope (TR 200, VWR International, Leuven, Belgium) at four-fold magnification. An initial viability value of more than 90 percent was considered necessary to perform the experiments.

Nanoparticle suspensions of the samples were added into the wells of a 96-well plate to achieve final concentrations of 0.0125%, 0.025%, 0.05%, and 0.1% *w*/*w* of *L. micranthus*. Afterwards, 10 µL of nematode suspension was added to each well and the final volume in each well was adjusted to 100 µL with phosphate buffered saline (PBS) (pH = 7.4). PBS was used in the negative control and ethanol in the positive control. Viability was determined at 0 h and 24 h by counting the live and dead nematodes, whereby 50 µL of hot water (50 °C) was added to each well to stimulate the nematodes for the 24 h reading [9]. Each experiment was performed independently in triplicate and on three different occasions (*n* = 9).

## 3. Statistical Analysis

All data (antimicrobial, antifungal, and nematicidal activities) were expressed as the standard error of the mean (±SEM). For nematicidal activity, data comparisons were performed using One-way analysis of variance (ANOVA), and post hoc analysis was carried out by the Student Newman-Keuls (SNK) test. For antimicrobial and antifungal activities, data were analysed by two-way ANOVA, Bonferroni post hoc test. GraphPad Prism (Version 5.03, GraphPad Software, USA) was used for data analysis and to generate charts. Statistical significance was set at * *p* < 0.05, ** *p* < 0.01, and *** *p* < 0.001.

## 4. Results and Discussion

### 4.1. Production and Characterisation of Nanosized Materials

**Figure 2:**
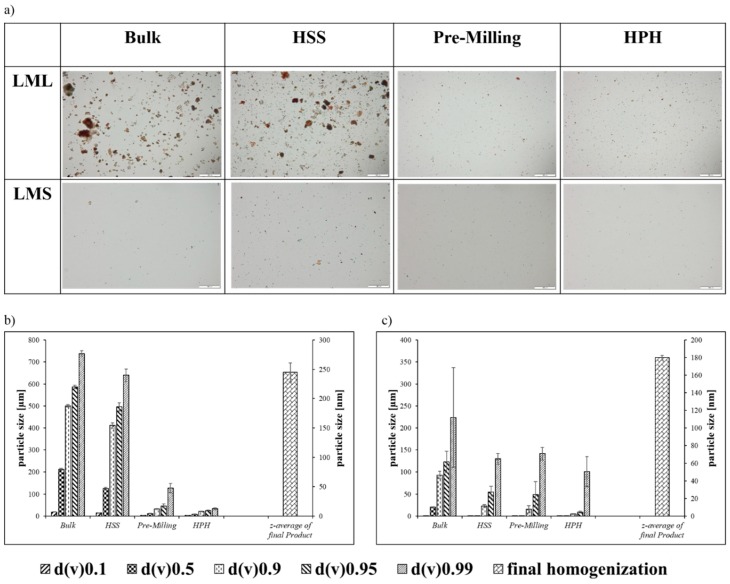
(**a**) Microscopic view of nanosizing from bulk material of *L. micranthus* leaf (LML) and *L. micranthus* stem (LMS) to high speed stirring (HSS) and high-pressure homogenization (HPH)-processed nanosized material. The micrographs are at 200-fold magnification with a scale bar of 100 µm. The characterization of samples using Laser Diffraction (LD) and Photon Correlation Spectroscopy (PCS) techniques is presented for (**b**) *L. micranthus* leaf (LML) samples and (**c**) *L. micranthus* stem (LMS) samples. The average size of the particles shown through these graphs demonstrates the effectiveness of the procedure where final sizes achieved for leaf samples are around 245 nm, and those of stem samples are around 180 nm. Z-average is the average of particles sizes and corresponds with the right y-axis of nanometer scale.

Nanoparticles produced from the leaves and stems of the African mistletoe parts varied from each other and the results are presented in Figure 2a–c. The results of the Mastersizer analysis indicated that there are still larger particles present in the final suspension of leaves and stem nanoparticles, which could be viewed by microscopy, yet these larger particles constituted less than 10% of the overall amount of nanoparticles in these suspensions (Figure 2a). The *Loranthus micranthus* leaves (LML) sample, consisting of larger particles, at the end of the milling procedure, initially showed a better size reduction response when compared to the *Loranthus micranthus* stem (LMS) sample. This response unfortunately, can only be seen during the high speed stirring (HSS) and initial homogenization steps, whereas it was difficult to break the leaf sample into smaller particles of less than 245 nm in diameter employing the high-pressure homogenization (HPH), most probably because the leaf particles were rather elastic when compared to the stem particles. The smallest size achieved for the leaf particles was 245 nm (Figure 2b). An attempt to break up these particles further by applying additional cycles at 1500 bar pressure agglomerated the samples irreversibly. The sample of the stem, in contrast, was fairly brittle and a higher pressure could be applied, resulting in a size reduction of particles to around 180 nm (Figure 2c).

### 4.2. Antimicrobial Activity

**Figure 3:**
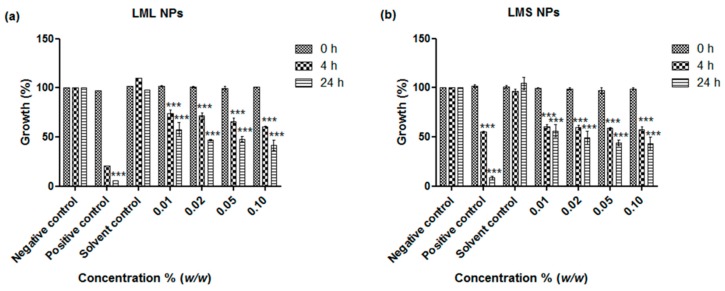
Influence of nanosized (**a**) *L. micranthus* leaf nanoparticles (LML NPs) and (**b**) *L. micranthus* stem nanoparticles (LMS NPs) on the growth of *E. coli*. Values represent mean ± SD *** *p* < 0.001. See text for experimental details.

**Figure 4:**
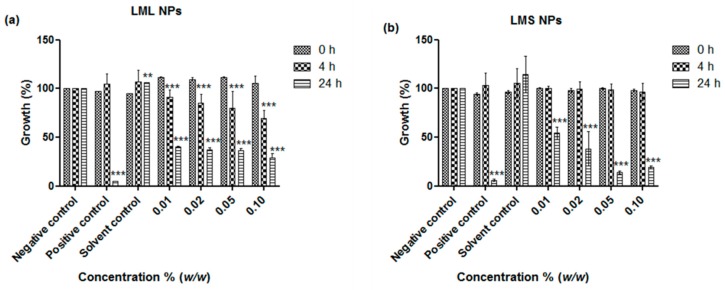
Influence of nanosized (**a**) *L. micranthus* leaf nanoparticles (LML NPs) and (**b**) *L. micranthus* stem nanoparticles (LMS NPs) on the growth of *S. carnosus.* Values represent mean ± SD ** *p* < 0.01 and *** *p* < 0.001. See text for experimental details.

The antimicrobial activities of the nanosized suspensions of the samples were determined in the assays of *E. coli* and *S. carnosus*, representing Gram-negative and Gram-positive bacteria, respectively. A strong and concentration-dependent inhibition of the growth of both *E. coli* and *S. carnosus* has been evident (Figure 3 and Figure 4). Even at a concentration of just 0.01% *w*/*w* of particles in media, both the nanoparticles obtained from the stems and the ones obtained from the leaves exhibited statistically highly significant antimicrobial activity against *E. coli* and *S. carnosus*, whereby the stem sample exhibited a higher activity when compared to the leaf sample. At higher particle concentrations, i.e., 0.05% and 0.1% *w*/*w*, the inhibition was 86% and 82%, respectively, as compared to the cocktail of standard antibiotics (penicillin, streptomycin, and amphotericin B) employed as the positive control, which inhibited the growth of *S. carnosus* by 94% (Figure 4b). These findings are rather revealing since traditional healers often use crude extracts of *L. micranthus* in the management of diarrhea, dysentery, and treatment of sores, boils, and open wounds, i.e., infections associated with a spectrum of Gram-negative and Gram-positive bacteria [4,10,11]. Besides confirming that the mistletoe and its ingredients exhibit a significant and probably broader antibacterial activity, our studies also demonstrate that nanosizing is well posed to unlock this potential in a simple, yet effective manner, without any cumbersome extractions and without producing any waste.

**Figure 5:**
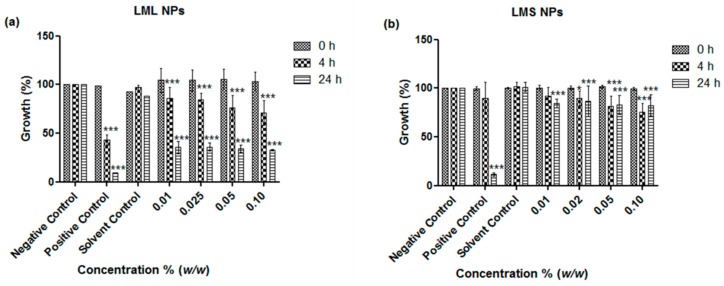
Influence of nanosized (**a**) leaves (LML NPs) and (**b**)stem (LMS NPs) of *L*. *micranthus* on the growth of *C. albicans*. Values represent mean ± SD *** *p <* 0.001. See text for experimental details.

**Figure 6:**
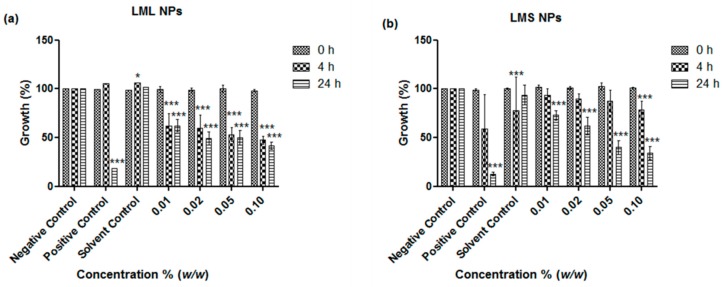
Influence of nanosized (**a**) *L. micranthus* leaf (LML NPs) and (**b**) *L. micranthus* stem nanoparticles (LMS NPs) on the growth of *S. cerevisae.* Values represent mean ± SD *** *p <* 0.001. See text for experimental details.

The remarkable activity of the nanosized samples of the leaves and stems against *E. coli* and *S. carnosus* by far exceeds the activities previously observed for similar nanosized materials derived from other plants, such as tomato stem and Maltese Mushroom [7,8,12]. Since KuntaMedizinmannwilli also traditionally employs mistletoe to treat the very same, the activity of the nanosuspensions against *C. albicans* and, as another model organism, non-pathogenic *S. cerevisiae*, were investigated. In contrast to the pronounced activity against the two selected strains of bacteria, the activity of the nanosuspensions against the two yeasts was rather modest, especially in the case of the stem-derived materials and *C. albicans* (Figure 5 and Figure 6). Some notable inhibition of growth could be achieved at higher concentrations of samples based on the leaves, and in the case of leaf-derived nanosuspensions and *C. albicans*, this inhibition was 66% at a concentration of 0.1% *w*/*w* as compared to the 90% inhibition achieved with the common fungicide ketoconazole, which served here as the positive control (Figure 5a). Once more, these findings are in good agreement with earlier studies which have shown that the crude extract of mistletoeleaves inhibits the growth of *C. albicans*, *Aspergillus* species, and *Pencillium* species, which are causative agents of infectious diseases such as candidiasis, respiratory mycosis, vaginosis, and pelvic inflammatory disease [13]. As in the case of bacteria, possible applications in the treatment of these human infectious diseases are feasible. Bearing in mind that access to modern antibiotics is often limited in developing countries, and home-grown extractions are sometimes not really entirely kosher, nanosizing the mistletoe to yield an—initially sterile because of high pressure involved -nanosuspension for direct applications may provide an attractive alternative, especially in less dramatic gastrointestinal and topical infectious diseases [14,15,16]. The notable activity against *S. cerevisiae* is also of interest as it may, in the future, allow wider chemogenetic phenotype profiling in yeast mutants in order to elucidate possible modes of action underlying this toxicity. 

**Figure 7:**
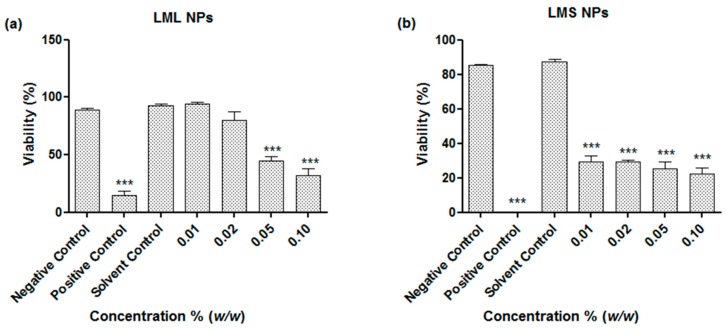
Influence of nanosized (**a**) *L. micranthus* leaf nanoparticles (LML NPs) and (**b**) *L. micranthus* stem nanoparticles (LMS NPs) on the viability of *S. feltiae*. Values represent mean ± SD *** *p* < 0.001. See text for experimental details.

Compared to other medically interesting plants and herbs which are also amenable to nanosizing yet cannot be harvested sustainably in large quantities, such as the Maltese Mushroom (*Cynamoriumcoccinium*), the African mistletoe is a ubiquitous weed which can be accessed locally, easily, in large quantities, and without causing any damage to diversity or the environment [12]. As a consequence, a large(r) scale application of this material is possible, for instance, in the fields of eco-friendly Agriculture. Here, pathogenic bacteria and fungi, but also nematodes, play an important role. The activity of the mistletoe derived nanosuspensions against the agricultural model nematode *S. feltiae* has therefore been investigated (Figure 7). 

As may be expected, the nanosuspensions investigated were also active against this model nematode. Once again, the best activity was noted for the stem-derived suspensions, which resulted in a reduction of viability to around 30% at concentrations as low as 0.01% *w*/*w*. The suspensions based on the leaves were also active, albeit only at higher concentrations, i.e., at 0.05% *w*/*w* and above.

As discussed already, it is likely that the multitude of natural compounds like alkaloids, polyphenols, and phenolics glycosides found in the stems and leaves of the mistletoe is responsible for this pronounced toxicity. These active ingredients, especially alkaloids, possess antimicrobial properties which explain the enhanced activity of *L*. *micranthus* nanosuspension against bacteria. Future studies will be needed to identify the exact nature and identification of these molecules, and their release properties from the nanoparticles, which will differ significantly from extractions with water or organic solvents. Such studies will also be needed to address the underlying modes of action, the selectivity and specificity, and the possible side effects associated with the compounds released from nanomilled plants. Furthermore, the question of nanotoxicity, which may indeed play a critical role in the activity observed against the multicellular nematodes, needs to be considered before any practical applications, either in the fields of Medicine or Agriculture, can be envisaged. An investigation into the effects of these nanoparticles NPs on human organism (cell lines) is ongoing in our laboratory and this will therefore be of public importance. As for practical applications, the medium- and long-term stability of the nanosuspensions, possible aggregation, degradation, or fouling of the materials and alternatives, such as freeze-drying and resuspension as part of the so-called NaLyRe sequence, need to be investigated [7]. Once such studies have been conducted successfully, possible applications as antimicrobial agents in humans and as green phyto-protectants in ecologically friendly agriculture can be envisioned.

## 5. Conclusions

Our studies with nanosized leaves and stems of the African mistletoe have shown that such nanosuspensions can be prepared easily and exhibit pronounced antimicrobial and nematicidal activities, which at higher particle concentrations of 0.1% (*w/w*), compete well with those of established antibiotics and fungicides. This study has provided an insight into the antifungal and antibiotics efficacy of herbal nanosized materials with an emphasis on *L. micranthus*.
